# Supplementary material for: The association between salivary amylase gene copy number and enzyme activity with type 2 diabetes status
Source: PLoS One. 2025 Jul 2;20(7):e0324660. doi: 10.1371/journal.pone.0324660 (PMC12221092; doi:10.1371/journal.pone.0324660)
Supplement: S1 Fig — Plot illustrating the plasma glucose and insulin responses during a mixed meal tolerance test at 0, 15, and 30 minutes. The figure displays three diabetes status groups: orange–individual with no T2D/prediabetes (n = 1); green–individuals with prediabetes (n = 4); blue–individuals with T2D (n = 13). Mixed meal tolerance test data were not available for one participant with T2D. (DOCX) [file pone.0324660.s001.docx]

**Mixed meal tolerance test in participants with self-reported type 2 diabetes or prediabetes (T2D/prediabetes) to confirm glucose dysregulation.**

A mixed meal tolerance test (MMTT) was performed on the T2D/prediabetes participants to record their glucose and insulin responses to a standardized liquid meal containing predetermined amounts of macronutrients. The MMTT was conducted following an overnight fast of at least 8 hours. Participants were instructed to consume a standard liquid meal, Boost Original Nutritional Drink, in 10 minutes. Blood samples were collected at baseline (0 min), 15 minutes, and 30 minutes after the ingestion of Boost. The blood samples were collected in serum separator tubes and centrifuged immediately after collection to obtain serum for further analysis. **Figure S1** shows participants’ glucose and insulin responses during the MMTT. The plots show distinct patterns in the glucose and insulin responses among participants with T2D, prediabetes, and without T2D/prediabetes.


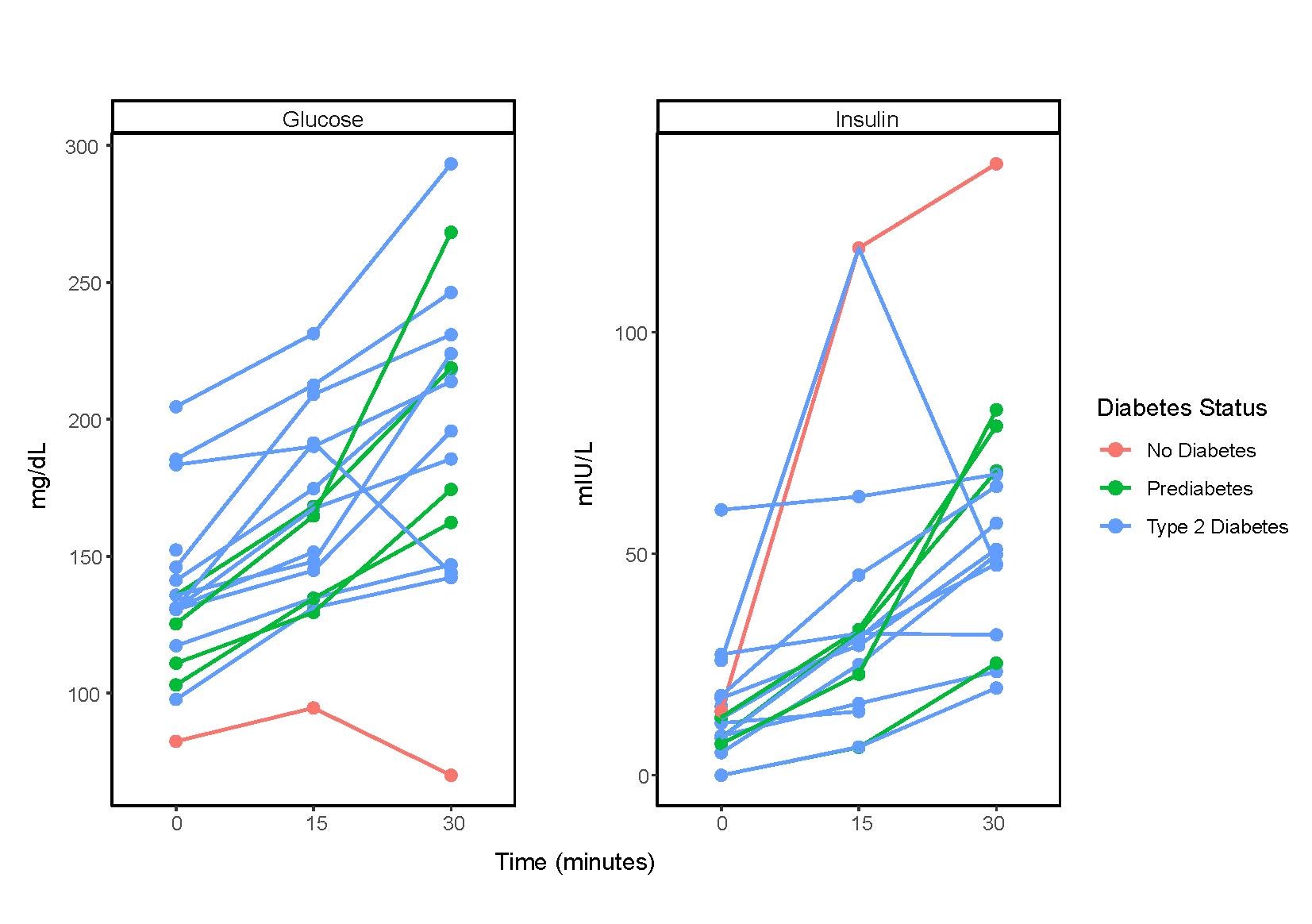


**Fig S1. Mixed meal tolerance test in participants with type 2 diabetes or prediabetes.** Plot illustrating the plasma glucose and insulin responses during a mixed meal tolerance test at 0, 15, and 30 minutes. The figure displays three diabetes status groups: orange–individual with no T2D/prediabetes (n = 1); green–individuals with prediabetes (n = 4); blue–individuals with T2D (n = 13). Mixed meal tolerance test data were not available for one participant with T2D.
