# Supplementary material for: The association between salivary amylase gene copy number and enzyme activity with type 2 diabetes status
Source: PLoS One. 2025 Jul 2;20(7):e0324660. doi: 10.1371/journal.pone.0324660 (PMC12221092; doi:10.1371/journal.pone.0324660)
Supplement: S1 Table — This table shows the mean and standard deviation of the participant demographics. The healthy controls (n = 76) included individuals who submitted up to four saliva samples at different times of the day. The T2D/prediabetes group (n = 18) includes individuals who submitted up to two saliva samples. (DOCX) [file pone.0324660.s002.docx]

|  | **Control (n = 76)** | **T2D/prediabetes (n = 18)** | **P-value** |
| --- | --- | --- | --- |
| Age in years | 24.55 ± 4.48 | 65.3 ± 11.17 | <0.001 |
| Sex (%) | Female - 68% | Female – 37.5% |  |
|  | Male – 32% | Male – 62.5% | 0.04 |
| *AMY1* CN | 7.70 ± 2.8 | 6.37 ± 2.48 | 0.07 |
| SAA | 112.6 ± 86.5 | 159.25 ± 160.98 | 0.11 |

**Table S1.** **Table comparing individuals with T2D/prediabetes to those without T2D/prediabetes (controls).** This table shows the mean and standard deviation of the participant demographics. The healthy controls (n = 76) included individuals who submitted up to four saliva samples at different times of the day. The T2D/prediabetes group (n = 18) includes individuals who submitted up to two saliva samples.
