# Supplementary material for: The association between salivary amylase gene copy number and enzyme activity with type 2 diabetes status
Source: PLoS One. 2025 Jul 2;20(7):e0324660. doi: 10.1371/journal.pone.0324660 (PMC12221092; doi:10.1371/journal.pone.0324660)
Supplement: S2 Table — (DOCX) [file pone.0324660.s003.docx]

| Formula: lm(*AMY1* CN_ddPCR ~ *AMY1* CN_qPCR)  Coefficients:  Estimate Std. Error t value Pr(>\|t\|)  (Intercept) 0.02093 0.18261 0.115 0.909  AMY1CN_q_PCR 0.91711 0.02128 43.098 <2e-16 ***  ---  Signif. codes: 0 ‘***’ 0.001 ‘**’ 0.01 ‘*’ 0.05 ‘.’ 0.1 ‘ ’ 1  Residual standard error: 0.9192 on 208 degrees of freedom  Multiple R-squared: 0.8993, Adjusted R-squared: 0.8988  F-statistic: 1857 on 1 and 208 DF, p-value: < 2.2e-16 |
| --- |

**Table S2. R output for linear regression to test the association between *AMY1* copy number values determined by qPCR and ddPCR.**
