# Supplementary material for: The association between salivary amylase gene copy number and enzyme activity with type 2 diabetes status
Source: PLoS One. 2025 Jul 2;20(7):e0324660. doi: 10.1371/journal.pone.0324660 (PMC12221092; doi:10.1371/journal.pone.0324660)
Supplement: S3 Table — (DOCX) [file pone.0324660.s004.docx]

| Formula: ICC(Dataframe [, c(AMY1_d_PCR, AMY1_q_PCR)], model = “twoway”, type = “consistency”, unit = “single”, conf.level = 0.95)  Subjects (sample#) = 210  Raters = 2  ICC(C,1) = 0.948 |
| --- |

**Table S3. R output for the intraclass correlation coefficient (ICC) to assess consistency between qPCR and ddPCR.**
