# Supplementary material for: The association between salivary amylase gene copy number and enzyme activity with type 2 diabetes status
Source: PLoS One. 2025 Jul 2;20(7):e0324660. doi: 10.1371/journal.pone.0324660 (PMC12221092; doi:10.1371/journal.pone.0324660)
Supplement: S4 Table — B, C) R output for linear mixed models testing association between SAA and time of saliva sample collection in B) morning samples only and C) afternoon samples only. (DOCX) [file pone.0324660.s005.docx]

| A. Formula: log(SAA) ~ time of saliva collection + (1 \| participant_id)    Random effects:  Groups Name Variance Std.Dev.  participant_id (Intercept) 0.4919 0.7013  Residual 0.1751 0.4184  Number of obs: 263, groups: participant_id, 76  Fixed effects:  Estimate Std. Error df t value Pr(>\|t\|)  (Intercept) 3.34757 0.22754 255.61340 14.712 < 2e-16 ***  Time of saliva collection 0.08580 0.01684 220.37669 5.094 7.52e-07 ***  ---  Signif. codes: 0 ‘***’ 0.001 ‘**’ 0.01 ‘*’ 0.05 ‘.’ 0.1 ‘ ’ 1 |
| --- |

| B. Saliva samples collected in the morning:  Formula: log(SAA) ~ time of saliva collection (morning samples) + (1 \| participant_id)  Random effects:  Groups Name Variance Std.Dev.  participant_id (Intercept) 0.4859 0.6971  Residual 0.1886 0.4343  Number of obs: 111, groups: participant_id, 51  Fixed effects:  Estimate Std. Error df t value Pr(>\|t\|)  (Intercept) 2.43678 0.63128 96.38994 3.860 0.000205 ***  time_numeric 0.17961 0.05938 93.08908 3.025 0.003216 **  ---  Signif. codes: 0 ‘***’ 0.001 ‘**’ 0.01 ‘*’ 0.05 ‘.’ 0.1 ‘ ’ 1 |
| --- |

| C. Saliva samples collected in the afternoon:  Formula: log(SAA) ~ time of saliva collection (afternoon samples) + (1 \| participant_id)    Random effects:  Groups Name Variance Std.Dev.  participant_id (Intercept) 0.4648 0.6818  Residual 0.1414 0.3760  Number of obs: 152, groups: participant_id, 63  Fixed effects:  Estimate Std. Error df t value Pr(>\|t\|)  (Intercept) 3.41428 0.61089 130.42241 5.589 1.28e-07 ***  Time of saliva collection 0.08503 0.04302 127.25596 1.976 0.0503 .  ---  Signif. codes: 0 ‘***’ 0.001 ‘**’ 0.01 ‘*’ 0.05 ‘.’ 0.1 ‘ ’ 1 |
| --- |

**Table S4. A) R output for linear mixed regression testing association between SAA and the time of saliva collection during the day using samples collected in the morning and in the afternoon. B,C) R output for linear mixed models testing association between SAA and time of saliva sample collection in B) morning samples only and C) afternoon samples only.**
