# Supplementary material for: The association between salivary amylase gene copy number and enzyme activity with type 2 diabetes status
Source: PLoS One. 2025 Jul 2;20(7):e0324660. doi: 10.1371/journal.pone.0324660 (PMC12221092; doi:10.1371/journal.pone.0324660)
Supplement: S5 Table — (DOCX) [file pone.0324660.s006.docx]

| Formula: log(SAA) ~ time of saliva collection + time_of_day (morning or afternoon) + time of saliva collection * day_phase (morning or afternoon) + 1\|participant  Random effects:  Groups Name Variance Std.Dev.  participant_id (Intercept) 0.4946 0.7033  Residual 0.1733 0.4163  Number of obs: 263, groups: participant_id, 76  Fixed effects:  Estimate Std. Error df t value Pr(>\|t\|)  (Intercept) 3.64910 0.58799 225.90375 6.206 2.57e-09 ***  Time 0.06351 0.04137 219.49799 1.535 0.1262  time_of_dayMorning -1.16835 0.77551 215.72684 -1.507 0.1334  Time:time_of_dayMorning 0.10693 0.06370 213.53953 1.679 0.0947.  ---  Signif. codes: 0 ‘***’ 0.001 ‘**’ 0.01 ‘*’ 0.05 ‘.’ 0.1 ‘ ’ 1 |
| --- |

**Table S5. R output for linear mixed regression testing for the difference between rates of SAA change in the morning and the afternoon.**
