# Supplementary material for: The association between salivary amylase gene copy number and enzyme activity with type 2 diabetes status
Source: PLoS One. 2025 Jul 2;20(7):e0324660. doi: 10.1371/journal.pone.0324660 (PMC12221092; doi:10.1371/journal.pone.0324660)
Supplement: S6 Table — (DOCX) [file pone.0324660.s007.docx]

| Formula: log(SAA) ~ AMY1CN+1\|participant_id  Random effects:  Groups Name Variance Std.Dev.  participant_id (Intercept) 0.4807 0.6933  Residual 0.1997 0.4468  Number of obs: 300, groups: participant_id, 94  Fixed effects:  Estimate Std. Error df t value Pr(>\|t\|)  (Intercept) 3.29795 0.22036 89.75471 14.966 <2e-16 ***  AMY1 CN 0.15266 0.02769 89.17525 5.512 3.4e-07 ***  ---  Signif. codes: 0 ‘***’ 0.001 ‘**’ 0.01 ‘*’ 0.05 ‘.’ 0.1 ‘ ’ 1 |
| --- |

**Table S6. R output for linear mixed regression model testing for association between SAA and *AMY1* CN.**
