# Supplementary material for: The association between salivary amylase gene copy number and enzyme activity with type 2 diabetes status
Source: PLoS One. 2025 Jul 2;20(7):e0324660. doi: 10.1371/journal.pone.0324660 (PMC12221092; doi:10.1371/journal.pone.0324660)
Supplement: S7 Table — (DOCX) [file pone.0324660.s008.docx]

| Formula: log(SAA) ~ AMY1CN * health_status + time_of_day (morning vs afternoon) + Age + Sex + (1 \| participant_id)    Random effects:  Groups Name Variance Std.Dev.  participant_id (Intercept) 0.4090 0.6395  Residual 0.1818 0.4264  Number of obs: 285, groups: participant_id, 90  Fixed effects:  Estimate Std. Error df t value Pr(>\|t\|)  (Intercept) 3.027695 0.340772 81.847562 8.885 1.24e-13 ***  AMY1CN 0.131462 0.029137 79.265133 4.512 2.20e-05 ***  health_statust2d -1.885951 0.585926 91.615135 -3.219 0.00178 **  time_of_dayMorning -0.321545 0.067603 230.007694 -4.756 3.48e-06 ***  Age 0.018423 0.007928 83.277524 2.324 0.02257 *  SexMale -0.018291 0.165042 83.655175 -0.111 0.91202  AMY1CN:health_statust2d 0.225122 0.079389 92.383203 2.836 0.00562 **  ---  Signif. codes: 0 ‘***’ 0.001 ‘**’ 0.01 ‘*’ 0.05 ‘.’ 0.1 ‘ ’ 1 |
| --- |

**Table S7. R output for linear mixed regression model assessing the effect of type 2 diabetes status on the association between *AMY1* copy number and salivary amylase activity.**
